# In vivo role of capsular polysaccharide in *Mycoplasma mycoides*

**DOI:** 10.1093/infdis/jiy713

**Published:** 2018-12-12

**Authors:** Joerg Jores, Elise Schieck, Anne Liljander, Flavio Sacchini, Horst Posthaus, Carole Lartigue, Alain Blanchard, Fabien Labroussaa, Sanjay Vashee

**Affiliations:** 1 Institute of Veterinary Bacteriology, University of Bern, Switzerland; 3 Institute of Veterinary Pathology, University of Bern, Switzerland; 4 French National Institute for Agricultural Research, Villenave d’Ornon, France; 5 University of Bordeaux, UMR 1332 de Biologie du Fruit et Pathologie, Villenave d’Ornon, France; 2 International Livestock Research Institute, Nairobi, Kenya; 6 J. Craig Venter Institute, Rockville, Maryland

**Keywords:** *Mycoplasma mycoides*, *M. mycoides* subsp*. capri*, carbohydrate, glycan, galactofuranose, virulence factor, vaccine, CPS

## Abstract

Capsular polysaccharides have been confirmed to be an important virulence trait in many gram-positive and gram-negative bacteria. Similarly, they are proposed to be virulence traits in minimal *Mycoplasma* that cause disease in humans and animals. In the current study, goats were infected with the caprine pathogen *Mycoplasma mycoides* subsp. *capri* or an engineered mutant lacking the capsular polysaccharide, galactofuranose. Goats infected with the mutant strain showed only transient fever. In contrast, 5 of 8 goats infected with the parental strain reached end-point criteria after infection. These findings confirm that galactofuranose is a virulence factor in *M. mycoides*.

## Teaser

Many *Mycoplasma* species have been shown to possess capsular polysaccharides (CPSs). We challenged goats with a CPS-defective or its CPS-possessing parental *Mycoplasma mycoides* strain. The CPS-defective strain was attenuated, demonstrating that the CPS consisting of galactofuranose is a virulence trait.

*Mycoplasma* species are economically important pathogens, especially in the veterinary sector. However, the mechanisms driving their pathogenicity and specifically their virulence traits remain largely speculative and based on in vitro data. Most available *Mycoplasma* vaccines have shortcomings, such as low efficacy and short duration of immunity. The development of next generation *Mycoplasma* vaccines will benefit greatly from knowledge of host-pathogen interactions. Capsular polysaccharides (CPSs) have been proposed as possible *Mycoplasma* virulence factors for >40 years [1]. They are at the interface between the bacterium and its host and constitute the first line of defense against the immune system.

Based on in vitro experiments, it has been suggested that the *Mycoplasma gallisepticum* capsule is involved in adhesion and virulence [2], the exopolysaccharides of *Mycoplasma pulmonis* protect against the complement system [3], CPS of *Mycoplasma dispar* down-regulates macrophage function [4], and purified CPS of *Mycoplasma ovipneumoniae* induces an inflammatory response through Toll-like receptor signaling in airway epithelial cells [5]. The exopolysaccharides of members of the “*Mycoplasma mycoides* cluster” have been characterized [6] and free CPS of *M. mycoides* induces the production of interleukin 10 in bovine macrophages, which acts as an anti-inflammatory [7].

Schieck et al [8], using synthetic biology tools, have shown that the CPS of *M. mycoides* subsp. *capri* modulates host-pathogen interactions in vitro. In the current study, we tested whether the CPS of *M. mycoides* constitutes a virulence trait. Using a caprine challenge model, we compared the outcomes of experimental infections with a highly virulent *M. mycoides* subsp. *capri* strain (GM12::YCpMmyc1.1) or its CPS-lacking mutant derivative (GM12::YCpMmyc1.1-*Δglf*). Goats were infected with either strain, and clinical parameters and pathomorphological changes were evaluated. Attenuation was shown for GM12::YCpMmyc1.1-*Δglf*, demonstrating a role of CPS as a virulence factor for this important group of mycoplasmas.

## METHODS

### Mycoplasma Strains

In the current study, we used *Mycoplasma mycoides* subsp. *capri* GM12 and its derivatives GM12::YCpMmyc1.1 [9] and GM12::YCpMmyc1.1-*Δglf* [8]. Strains were cultivated at 37°C in Difco™ PPLO Broth (Catalogue No. 255420) supplemented with 20% heat-inactivated horse serum (Sigma; catalogue No. H1138) 0.5% glucose, 0.03% penicillin G, 0.5 g/L thallium acetate and 0.9 g/L yeast extract to early logarithmic phase, aliquoted and stored at −80°C. Immediately before infection, the vials were thawed and the concentration of *Mycoplasma* was adjusted to 10^8^ or 10^9^ colony-forming units (CFUs)/mL using broth culture medium. Bacterial concentrations were confirmed using the 10-fold titration method followed by plating on Difco™ PPLO Agar supplemented as above for quantifying CFUs per milliliter, using 2 aliquots for each strain.

### Animal Experiment Setup

All protocols in the current study were designed and performed in strict accordance with Kenyan and American laws concerning animal experimentation and were approved by the institutional animal care and use committees at both institutions (J. Craig Venter Institute and International Livestock Research Institute [ILRI]; Institutional Animal Care and Use Committee reference 2016.06). Sixteen male outbred goats (*Capra aegagrus hircus*), 1–2 years of age and randomly selected at the ILRI ranch (Kapiti) east of Nairobi, were transferred to the ILRI campus in Nairobi and kept under quarantine for 2 months. After arrival at the campus, all goats were dewormed twice with levamisole and treated prophylactically with imidocarb to prevent babesiosis and anaplasmosis. They were vaccinated against anthrax and blackleg (Blanthax; Cooper), foot-and-mouth disease (FOTIVAX™; Kevevapi), and Peste des Petits Ruminants (live attenuated strain Nigeria 75/1). All animals tested negative for antibodies against contagious caprine pleuropneumonia (CCPP), using a competitive enzyme-linked immunosorbent assay (IDEXX Laboratories).

Two weeks before experimental infection, all animals were transferred to the animal biosafety level 2 unit. They were allowed to move freely within the unit, had ad libitum access to water and hay, and received additional pellets each morning. Three veterinarians monitored the animals’ health status throughout the experiment. Rectal temperature, oxygen blood saturation, pulse rate, and respiration rate were measured daily in the morning hours, using a GLA M750 thermometer (GLA Agricultural Electronics), VE-H100B oximeter (Edan), and a Littmann Classic II stethoscope, respectively. All animals were observed for at least 20 minutes thrice daily, and behavioral observations were recorded throughout the study. They were weighed and venous blood samples were obtained thrice weekly, and nasal swab samples (Flocked swabs; Copan) were obtained twice weekly. Swab samples were transferred into cryotubes filled with media and stored at −80°C until further processing. On the day of infection, the 2 groups were inoculated transtracheally by needle puncture 5–10 cm distal of the larynx with 10^8^ CFUs of the respective strains *M. mycoides* subsp. *capri* GM12::YCpMmyc1.1 and GM12::YCpMmyc1.1-*Δglf*.

Seven days after infection, the goats were given a booster infection of 10^9^ CFUs of the respective strain transtracheally. Animals were euthanized if they had a rectal temperature of >40.5°C for >3 consecutive days, moderate to severe pain or distress, weight loss >10% within 7 days, or a respiration rate >50/min for >3 days. The remaining animals in the group that had received *M. mycoides* subsp. *capri* GM12 YCpMmyc1.1 were euthanized at 35 days after infection. All 8 animals in the group that had received *M. mycoides* subsp. *capri* GM12 YCpMmyc1.1-*Δglf* were challenged by 10^9^ CFUs of *M. mycoides* subsp. *capri* GM12 at 35 days after infection transtracheally. Monitoring and sampling continued as described above for up to 63 days after infection, when the remaining animals were euthanized. Euthanasia was performed by intravenous injection of Lethabarb Euthanasia Injection (Virbac) (200 mg/kg body weight).

### Pathomorphological and Histological Analysis

A complete necropsy was performed on all animals. Tissue samples were fixed in 10% buffered formalin for 72 hours and subsequently processed routinely for paraffin embedding. Tissue was cut in 3-µm sections, which were stained routinely with hematoxylin-eosin and evaluated by a board-certified pathologist (H.P).

### Microbiology

Venous blood samples, lung samples, carpal joint fluid, and pleural fluid specimens were subjected to isolation of *Mycoplasma,* as described elsewhere [10], using Mycoplasma Liquid Medium (Mycoplasma Experience LTD). Lung samples and pleural fluid were screened for *Pasteurella* spp. and *Mannheimia* spp. using standard methods [11].

### Statistical Analysis

Log-rank (Mantel-Cox) tests were performed to determine *P* values for survival rates of the 2 groups, comparing their reaching of end-point criteria; GraphPad Prism software was used (version 7.0d).

## RESULTS

### Galactofuranose as a Virulence Factor

Two groups of 8 animals each were inoculated with either GM12::YCpMmyc1.1-*Δglf* or its parental strain GM12::YCpMmyc1.1. Three days after infection, 1 animal (CM154) in the group receiving GM12::YCpMmyc1.1 presented with moderate pyrexia (>39.5°C to 40.5°C), followed by 5 additional animals within the next 10 days (CM158, CM159, CM181, CM191, and CM197). All of these animals except 1 (CM191) had severe pyrexia (body temperature, >40.5°C) on at least 1 day. Two of them (CM154 and CM158) had severe pyrexia for >3 consecutive days, which had been defined as an end-point criterion. Depression and anorexia were observed in the animals. Two in this group were euthanized because they had lost >10% of their body weight within 7 days, and 5 showed signs of depression. Severe arthritis developed in 1 animal (CM181) in the GM12::YCpMmyc1.1 group, which was also euthanized. The 3 animals that did not reach end-point criteria showed various clinical signs, such as fever and depression (Table 1), and were euthanized at 35 days after infection. The animals in this group had the same lesions (Supplementary Table 1) seen in another caprine challenge experiment using GM12 (J. J. et al, unpublished findings), except for animal CM181, which also showed arthritic lesions (Table 1).

**Table 1.** Clinical Observations 0–35 Days After Infection for the Group of Animals Receiving the Parental Strain GM12::YCpMmyc1.1^a^

**Animal No.**	**Time After Infection, d**				
	**Bacteremia**	**Depression,**	**High Fever Peaks** ^b^	**Arthritis**	**Euthanasia** ^c^
CK045	NO	NO	NO	NO	No
CM047	NO	NO	NO	NO	No
CM154	7 (10^3^ CFUs/mL)	4–8	5–8	NO	8
CM158	7 (10^2^ CFUs/mL)	7–9	6–9	NO	9
CM159	NO	8–10	9	NO	13
CM181	NO	8, 13, 19	12	18–20	20
CM191	NO	NO	NO	14	No
CM197	11 (10^2^ CFUs/mL)	9–12	11	NO	12

Abbreviations: CFUs, colony-forming units; NO, not observed.

^a^Findings for animals infected with the strain GM12::YCpMmyc1.1-Δglf are not displayed because that group did not show the clinical observations listed here.

^b^High fever peaks were defined as rectal temperatures >40.5°C.

^c^In animal euthanized before the end of the study (<35 days after infection).

**Figure 1.**
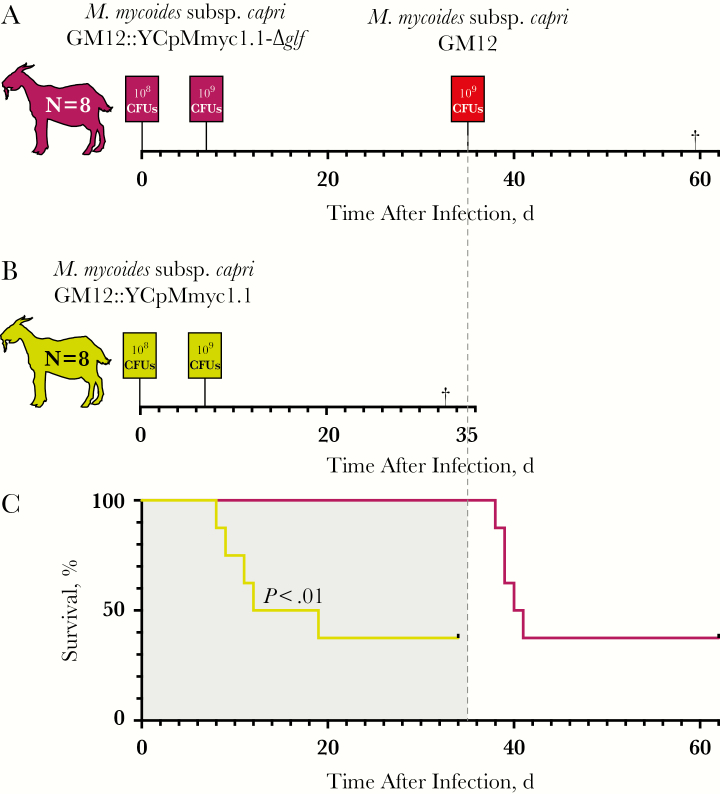
*A, B,* Setup of the in vivo trial. Two groups of outbred goats were infected with either GM12::YCpMmyc1.1-*Δglf* (*A*) or GM12::YCpMmyc1.1 (*B*) on the day of infection (day 0) and 7 days after infection. The group receiving GM12::YCpMmyc1.1 (*B*) was euthanized after reaching the end-point criteria or at 35 days after infection, if the did criteria were not reached. The other group (*A*) was challenged with GM12 at 35 days after infection. Abbreviations: CFUs, colony-forming units; *M. mycoides, Mycoplasma mycoides*. *C,* Survival rates. The *P* value represents the difference between the groups in reaching end-point criteria, determined using the log-rank (Mantel-Cox) test.

In the GM12::YCpMmyc1.1-*Δglf* group, the clinical picture was very different. Only 3 animals (CK044, CM122, and CM157) presented with moderate pyrexia during the follow-up period, and none with severe pyrexia. Coughing and nasal discharge were observed sporadically in both groups (Table 1). Respiration and pulse rates remained within the normal range throughout the study, although the latter was slightly higher as the animals entered the facilities and stabilized during the acclimatization week before infection. In total, 5 animals in the group infected with GM12::YCpMmyc1.1 reached end-point criteria and were euthanized. In contrast, none of the animals in the group infected with GM12::YCpMmyc1.1-*Δglf* reached end-point criteria (Figure 1). This difference was significant (*P* = .005), which shows that the mutant strain GM12::YCpMmyc1.1-*Δglf* is significantly attenuated. Therefore, we conclude that galactofuranose, present in the GM12::YCpMmyc1.1 but absent in GM12::YCpMmyc1.1-*Δglf*, contributes to virulence in *M. mycoides* subsp. *capri* in vivo.

### Insufficient Protective Immune Response Induced by GM12::YCpMmyc1.1-*Δglf* Infection

Because the clinical outcome of the infection differed significantly between the 2 groups of animals, we challenged the animals that received GM12::YCpMmyc1.1-*Δglf* to determine whether their infection induced a protective immune response. These animals were subjected to a homologous challenge 35 days after infection by transtracheal inoculation with *M. mycoides* subsp. *capri* GM12. In 5 of the 8 animals severe disease developed that required euthanasia between 39 and 42 days after infection; the severe disease included severe pyrexia, depression, and arthritis (Supplementary Table 2).

### Isolation of *Mycoplasma*

We have been able to isolate *M. mycoides* subsp. *capri* GM12::YCpMmyc1.1 in blood samples from animals CM154, CM158, and CM197, coinciding with severe pyrexia. Therefore, we inoculated serial dilutions of blood in liquid medium and confirmed the presence of the different strains using polymerase chain reaction (Supplementary Table 2). Bacterial titers ranged between 100 and 1000 CFUs/mL (Table 1). In contrast, we could not isolate GM12::YCpMmyc1.1-*Δglf* from any of the 8 animals inoculated with this mutant strain. However, GM12 was isolated in blood samples from the 3 animals that died 39–42 days after infection because of the homologous challenge with GM12 at 35 days after infection. Bacterial titers ranged between 10^4^ and 10^10^ CFUs/mL (Supplementary Table 2).

## DISCUSSION

The aim of the current study was to test the hypothesis that CPS in *Mycoplasma* is a virulence factor. Therefore, we took advantage of *M. mycoides* subsp. *capri,* which is amenable to precise genetic modification and has an infection model using its native host (J. J. et al, unpublished findings), to determine unambiguously the role of CPS in virulence in vivo. The results of this infection trial convincingly support the initial hypothesis that CPS is a virulence factor. None of the animals infected with the *glf* mutant (galactofuranose deficient) showed any clinical characteristics of severe disease, whereas 5 of the 8 infected with the parental strain GM12::YCpMmyc1.1 reached end-point criteria and were euthanized. In another animal trial, using 10^9^ CFUs of the *M. mycoides* subsp. *capri* GM12, the morbidity rate was 100%, and 100% of animals reached end-point criteria within 6 days after infection (J. J. et al, submitted).

It should be noted that the clinical picture of the group receiving GM12::YCpMmyc1.1 slightly differed from that of the group receiving GM12. This difference might be attributed to the different mode of infection represented by a superinfection or by the impact of integration of the YCp plasmid into the genome of GM12, though the latter possibility seems unlikely. Indeed, given the in vitro data Schieck et al [8] provided as part of the characterization of the mutant GM12::YCpMmyc1.1-*Δglf*, it cannot be ruled out that the slower growth time of the mutant strain was partly responsible for better bacterial clearance via the innate immune system. However, many highly virulent *Mycoplasma* species of the *M. mycoides* cluster [12], such as *Mycoplasma capricolum* subsp. *capripneumoniae* and *M. mycoides* subsp. *mycoides* have doubling times much longer than that of the mutant strain.

We tested whether the infection with the attenuated strain induced a protective immune response by conducting a homologous challenge. Only 3 of 8 animals, previously infected with the GM12::YCpMmyc1.1-*Δglf* and challenged with the GM12 strain, did not reach end-point criteria. This implies an insufficient immune response.

Our study findings encourage the development of a glycoconjugated vaccine for *Mycoplasma*. Initial data on a glycoconjugated vaccine approach for *M. mycoides* subsp. *mycoides* show some good prospects and support our findings [13]. The important role of CPS in *M. mycoides* requires further investigation as to exactly how this bacterium uses its enzymatic machinery as a virulence trait to identify key components that can be targeted for the rational design of efficient subunit vaccines.

## Supplementary Material

Supplementary Data

## Notes

Presented in part: 22nd Congress of the International Organization for Mycoplasmology, Portsmouth, New Hampshire, 9–12 July 2018.

## Notes

***Acknowledgments.* **We thank the animal handlers at the International Livestock Research Institute for their help, and we thank Pascal Sirand-Pugnet for fruitful discussions.

***Financial support.* **This work was supported by the National Science Foundation (grant IOS-1110151), the German Federal Ministry for Economic Cooperation and Development (contracts 81170269 and 81136800), the Consultative Group on International Agricultural Research (CGIAR) Research Program on Livestock and Fish) (CRP3.7), and the Centrum for International Migration (support to A. L.)

***Potential conflicts of interest.* **All authors: No reported conflicts. All authors have submitted the ICMJE Form for Disclosure of Potential Conflicts of Interest. Conflicts that the editors consider relevant to the content of the manuscript have been disclosed.
