# Supplementary material for: In vivo role of capsular polysaccharide in Mycoplasma mycoides
Source: J Infect Dis. 2018 Dec 12;219(10):1559–63. doi: 10.1093/infdis/jiy713 (PMC6473168; doi:10.1093/infdis/jiy713)
Supplement: Supplementary Data [file jiy713_suppl_supplementary_data.docx]

Additional file 1A: Pathology records of goats belonging to the two groups. Cave the group GM12::YCpMmyco1.1 was not challenged with wtGM12, while the other group received a homologous challenge at 35 dpi

| **Group** | **Date euthanized** | **Animal ID** | **Fibrinous Arthritis** | **Edema and inflammation in soft tissues at inoculation site with enlarged regional lymph nodes** | **Focal tracheitis at inoculation site** | **Broncho-pneumonia** | **Pleuritis** | **Histological findings** |
| --- | --- | --- | --- | --- | --- | --- | --- | --- |
| GM12::YCpMmyco1.1-*Δglf* | 42 dpi | CM044 | YES | YES | YES | YES |  | necrotizing tracheitis, necrosis and purulent inflammation around inoculation site, necrotizing lymphadenitis in tracheal lymph nodes, mild purulent bronchopneumonia |
|  | 39 dpi | CM050 |  | YES | YES |  | YES | necrotizing tracheitis, necrosis and purulent inflammation around inoculation site, necrotizing lymphadenitis in tracheal lymph nodes, necrotizing splenitis |
|  | 41 dpi | CM122 | YES | YES | YES |  | YES | necrotizing tracheitis, necrosis and purulent inflammation around inoculation site, necrotizing lymphadenitis in tracheal and peripheral lymph nodes, necrotizing splenitis |
|  | 63 dpi | CM128 | YES |  |  |  | YES | focal necrosis and granulomatous inflammation at inoculation site |
|  | 40 dpi | CM149 |  | YES | YES |  | YES | focal necrotizing tracheitis, necrosis and purulent inflammation at inoculation site |
|  | 63 dpi | CM157 |  |  |  |  | YES | no lesions |
|  | 63 dpi | CM178 |  |  |  |  |  | mild lymphocytic infiltration in tracheal submucosa at inoculation site |
|  | 40 dpi | CM187 | YES | YES | YES |  | YES | focal necrotizing tracheitis, necrosis and purulent inflammation at inoculation site, necrotizing lymphadenitis in tracheal lymph node |
| GM12::YCpMmyco1.1 | 35 dpi | CM045 | YES |  |  | YES |  | no lesions |
|  | 35 dpi | CM047 |  |  |  |  |  | no lesions |
|  | 8 dpi | CM154 |  | YES | YES |  |  | necrotizing tracheitis, necrosis and purulent inflammation around inoculation site, necrotizing lymphadenitis in tracheal lymph nodes |
|  | 9 dpi | CM158 |  | YES | YES |  |  | necrotizing tracheitis, necrosis and purulent inflammation around inoculation site, necrotizing lymphadenitis in tracheal lymph nodes |
|  | 13 dpi | CM159 | YES | YES | YES |  |  | necrotizing tracheitis, necrosis and purulent inflammation around inoculation site, necrotizing lymphadenitis in tracheal lymph nodes |
|  | 20 dpi | CM181 | YES | YES | YES |  |  | necrotizing tracheitis, necrosis and purulent inflammation around inoculation site, necrotizing lymphadenitis in tracheal lymph nodes |
|  | 35 dpi | CM191 |  |  |  |  |  | no lesions |
|  | 12 dpi | CM197 |  | YES | YES | YES | YES | necrotizing tracheitis, necrosis and purulent inflammation around inoculation site, necrotizing lymphadenitis in tracheal lymph nodes |

Additional file 1B: Clinical observations between 35-63 days post infection (35 dpi = homologous challenge with wtGM12)

| **Group** | **Animal ID (euthanized)** | **Bacteremia (CCU/ml)** | **Depression (No. of days)** | **High fever (>40.5C)** | **Arthritis** |
| --- | --- | --- | --- | --- | --- |
|  |  |  |  |  |  |
| GM12::YCpMmyco1.1-*Δglf* | CM044 (42 dpi) | 10^4^ (39 dpi), 10^6^ (42 dpi) | 3 | 39-42 dpi |  |
|  | CM050 (39 dpi) | 10^10^ (39 dpi) | 3 | 37-38 dpi | YES |
|  | CM122 (41 dpi) | 10^4^ (39 dpi) | 2 | 40-41 dpi | YES |
|  | CM128 (63 dpi) |  |  |  | YES |
|  | CM149 (40 dpi) | 10^6^ (39 dpi), 10^8^ (40 dpi) | 4 | 39 dpi | YES |
|  | CM157 (63 dpi) |  |  |  |  |
|  | CM178 (63 dpi) |  |  |  |  |
|  | CM187 (40 dpi) | 10^8^ (39 dpi), 10^8^ (40 dpi) | 3 | 38-40 dpi | YES |
